# “Better If They Laugh with Me than at Me”: The Role of Humor in Coping with Obesity-Related Stigma in Women

**DOI:** 10.3390/ijerph18157974

**Published:** 2021-07-28

**Authors:** Natalia Mazurkiewicz, Mariusz Lipowski, Jarosław Krefta, Małgorzata Lipowska

**Affiliations:** 1 Institute of Psychology, University of Gdańsk, 80-309 Gdańsk, Poland; psycholog.mazurkiewicz@gmail.com; 2 Department of Psychology, Gdańsk University of Physical Education and Sport, 80-336 Gdańsk, Poland; mariusz.lipowski@awf.gda.pl; 3 Creative Code Studio—Jarosław Krefta, 81-602 Gdynia, Poland; j.k.krefta@gmail.com

**Keywords:** obesity, coping with stress, stigmatization, humor style, body esteem

## Abstract

This study investigated the role of perceived stigmatization in the relationship between humor styles and coping with stress among young women suffering from stigma due to obesity. In the 21st century, obesity is an increasing global health issue with many physical and mental consequences for obese women. As a chronic stigmatizing disease, it requires that the affected individuals cope with social consequences; women with obesity are more prone to such consequences than men. Humor fosters the breaking of stereotypes and alleviating the consequences of stigmatization. A total of 127 young adult women (age *M* = 25.74, *SD* = 2.73) participated in the study (*n* = 54 with overfat and *n* = 73 with healthy fat). Participants filled out the Humor Styles Questionnaire, Perceived Stigmatization Questionnaire, and the Brief COPE Scale. Anthropometric data were gathered using a body composition analyzer. Results indicate that, when perceiving hostile behaviors toward themselves and using humor as a coping strategy, women with overfat select maladaptive styles of humor (i.e., self-defeating and aggressive styles). Women with overfat were also more likely to use humor as a coping strategy in difficult situations. Furthermore, none of the participants were satisfied with their body mass. At the same time, among women without obesity, a lack of compliments was not treated as a problem, even if they had high body fat.

## 1. Introduction

According to the World Health Organization (WHO), obesity is the most common metabolic disease, a global issue in the 21st century, and its prevalence is continually increasing [1]. In 2014, the Central Statistical Office of Poland reported that half of all adults (defined as persons aged over 15) in Poland were overweight [2], while, in 2019, 51% were overweight and 21% obese [3]. Worldwide data from 2016 placed Poland 67th in terms of the percentage of individuals with obesity (23.1%) [4]. A report prepared by the Parliamentary Bureau of Research indicates that the obesity rate continues to increase and is now one of the most common lifestyle-related health problems, posing a challenge to the health care system in Poland [5].

Obesity is not just a problem with a broad, worldwide scope—it also has many consequences for physical [6,7,8] and mental health [9,10,11]. Moreover, obesity lowers the quality of life [12] creating a vicious cycle of problems; for example, some types of depression can cause obesity, while in other situations, obesity can lead to depression [13,14]. This relation is stronger in women, for whom obesity is met with greater social censure than men. The body image of girls and women is a much more significant part of their “self” and has more bearing on their overall self-esteem than for men [15,16]. Women are also more prone to being overweight or obese than men [17], which has a far-reaching influence on their health, including in the context of reproduction—women with obesity are more at risk of gestational diabetes, preeclampsia, cesarian section, and neonatal morbidity [18]. Excess weight is a challenge for both the health system and the individual with obesity.

### 1.1. Obesity and Stigma

Stigma is one of the difficulties faced by overweight individuals. An individual who possesses an attribute that is socially stigmatized is themselves stigmatized and perceived as less valuable [19]. A stigma may be invisible or discreditable, and its disclosure may be at the discretion of the stigmatized person. However, obesity is chronic and discredit and thus visible. It is stigmatized in a multidimensional way, i.e., not just as a problem of one’s appearance or body but also as a problem of one’s character [19,20,21]. Body types outside the norms propagated by society and culture are met with discrimination and judgment [22]. Media all over the world promote slim, well-kept body types that are not attainable for every woman, increasing the risk of eating disorders [23,24,25]. Individuals with obesity experience rejection, animosity, and isolation and are perceived as less attractive, possessing inferior character, lazy, unsuccessful, and unintelligent [26,27,28,29]. People with obesity are judged and stigmatized by strangers, as well as their families and friends [30]. The "anti-fat" bias is common in healthcare facilities, which paradoxically increases the risk of obesity. Patients feel stigmatized and feel that they are receiving inferior care. Internalization of the weight stigma is often associated with high levels of alcohol consumption and psychoactive substances to cope also with stigma. [31]

Both stigma and body image depend to a large extent on the culture and customs regarding the understanding and perception of body types in a given region, as well as on an individual’s perception of other people’s opinions on body appearance [32,33]. Whether and to what extent obesity impairs social functioning and to what extent an individual needs to cope with the stigma depends on this perception and the image of self. 

### 1.2. Coping with Obesity-Related Stigma

Stigma in itself is associated with stress and the need to manipulate one’s situation or perceptions in order to reduce its consequences [19]. Satisfaction with one’s body image partially mediates the relationship between the amount of excess weight and depression and self-esteem, independently of gender [34]. Obesity poses an objective risk for increased stress. It is also subjectively perceived by women as a source of psychological stress and contributes to increased anxiety [35]. Stress can be both the cause and the result of obesity: eating is a common response to negative emotions or low mood [36,37]. 

Activities undertaken to cope with stress may also depend on one’s emotional well-being. A person may try to cope with obesity stigma by addressing the proximate cause of the stigma by losing weight or by taking pride in the condition and mobilizing others to prevent discrimination [38].

Women experience psychological discomfort related to their own appearance more often than men because their unattractive appearance is met with greater social disapproval. In both girls and women, self-image is more closely related to body image and affects self-esteem more than it affects their male counterparts [15,16]. Therefore, women with obesity, to cope with weight-related stigma, can use numerous maladaptive strategies, such as ignoring stigmatizing comments, extreme denial, involved self-criticism, and avoidance of distressing situations, or even overeating [38,39,40,41].

On the other hand, individuals with overweight are more likely to be presented in entertainment as engaging in unhealthy and stereotypical behaviors (e.g., eating or binging on unhealthy foods); referring to one’s own weight is met with laughter from audiences and obese individuals are a target of ridicule and humor [42]. Humor can serve to reduce tension and release repressed energy [43,44,45]. Humor and laughter can regulate one’s psychophysical balance, leading to reduced tension and psychosomatic problems, while also increase psychological well-being, and reduce chronic pain and stress [46,47,48]; it is also associated with one’s sense of happiness and even the ability to forgive [49]. Humor may facilitate the breaking of stereotypes about people who are ill or have some condition, such as obesity [50], and may even help to reduce stigmatization in case of revealing mental problems [51]; it can help to cope with loneliness [52], influence one’s mood when confronting negative stimuli [53], and increase one’s adaptive abilities in the face of stress at work [54]. According to a study by Abel [46], students qualified as people with a high sense of humor rated less stress and reported less current anxiety than those with a low sense of humor, despite having experienced a similar number of daily problems in the past two months. Humor is a tool through which an individual may express themselves, and it can be used interpersonally (focused on others, relational) or interpersonally (focused on oneself). It is a relatively stable personality trait, which can also be thought of as one’s characteristic style of humor [55,56,57]. Humor can function both adaptively and maladaptively. Martin et al. [56] identified affiliative humor and self-enhancing humor as being important to the well-being of the individual. While the other two, aggressive humor and self-defeating humor, are considered harmful and maladaptive.

Social disapproval of obesity and the associated sense of stigma (mainly in women) is associated with the need to seek mechanisms or ways to increase one’s social acceptance. Kruczek and Basińska [57] showed that individuals who use humor as a way of coping with stress perceived less stress. 

Summing up, a sense of humor used in difficult situations as a coping strategy enables handling obesity stigma difficulties. Women, who are more prone to social disapproval of excess body fat than men, must therefore use coping strategies.

Thus, the goal of the presented study was to investigate the role of perceived stigmatization as a mediator between humor style and preferred strategies of coping with stress among young women. 

The following hypotheses were made:

**Hypothesis 1.**  *The higher a person’s level of adaptive humor style is, the more adaptive their strategies of coping with stress (see Section 2.2.3. Procedure) will be; this relationship will be stronger in women with overfat than in women with healthy fat.*

**Hypothesis 2.**  *Perceived stigmatization will mediate the relationship between humor style and preferred strategies of coping with stress.*

## 2. Materials and Methods

### 2.1. Participants

A total sample of 127 females was recruited for this study. Data were collected between 2018 and 2019. We only invited women to participate in our research, because, for them, the weight-related stigma is of particular importance [58]. According to the research, body weight explains as much as 53% of the variance of attractiveness in women, while in men, only 13% of the variance [59]. Women in early adulthood (age: *M =* 25.74, *SD =* 2.73, min *=* 19, max = 30) were included in the study based on BMI, in accordance with the WHO classification [1]. Individuals with a BMI of ≥30 were included in the group with obesity (*n =* 54). In order to verify whether the aspect under examination is specific only for people with overfat, a control group (*n* = 73, with BMIs between 18.5 and 24.5) was also taken into account in the study. A body composition analyzer was used to perform the classifications.

### 2.2. Procedure

The recruitment procedure had two stages. During the first stage, 30 females participating in a larger project described elsewhere [60] who met the inclusion criteria for the study were recruited. During the second stage, females who met inclusion criteria for this research project were asked to invite acquaintances to participate, i.e., a non-random method of sample selection (“snowball sampling technique”), as our research group consisted of non-standard individuals [61]. Participants completed self-rated, paper-and-pencil questionnaires at home within two weeks or in a psychologist’s or dietician’s office, where the body mass measurement was later performed. 

The protocol of this study was approved by the Ethics Board for Research Projects at the Institute of Psychology, University of Gdansk, Poland (decision no. 12/2018). 

We collected several different kinds of information using the Humor Styles Questionnaire, the Perceived Stigmatization Questionnaire, and the Brief COPE Scale. Participants filled out also a short questionnaire with questions about age, ideal weight, height, presence of other chronic diseases. The objective body parameters of all participants were controlled by using a body composition analyzer. The data used for this study were part of a larger survey, and the questionnaires that formed this study took around 20 minutes to complete. 

#### 2.2.1. Humor Styles Questionnaire (HSQ)

The Humor Styles Questionnaire by Martin et al. [56], in its Polish adaptation by Hornowska and Charytonik [62], allows the assessment of style of humor. The questionnaire is composed of 32 items that form 4 subscales: *Self-enhancing humor, Aggressive humor, Affiliative humor*, and *Self-defeating humor*. For each item, participants assess on a 7-point scale how often the described humor-related situation applies to them. Cronbach’s alpha were as follows: *Self-enhancing humor* α = 0.66*, Aggressive humor* α = 0.64*, Affiliative humor* α = 0.65, and *Self-defeating humor* α = 0.75.

#### 2.2.2. Perceived Stigmatization Questionnaire (PSQ)

The Perceived Stigmatization Questionnaire by Lawrence et al. [63] measures the overall subjective sense of stigma. The questionnaire includes 21 items across 3 subscales: *Absence of friendly behavior, Confused/staring behavior, Hostile behavior*, and *Total Score*. Participants use a 5-point Likert-like scale to rate how often people behave in certain ways around them, where 1 indicates never, 5 indicates always, and 3 indicates sometimes. To develop a Polish version of the PSQ, with the author’s consent, the questionnaire was translated into Polish independently by an interpreter and a psychologist. After selecting the best Polish version, it was back-translated into English by a native speaker. Then, the quality of the translation was assessed by comparing the back translation with the original questionnaire. Cronbach’s alpha were as follows: *Absence of friendly behavior* α = 0.63; *Confused/Staring behavior* α = 0.70; *Hostile behavior* α = 0.77.

#### 2.2.3. Brief COPE Scale

Strategies of coping with stress behavior was determined by using the Brief COPE Scale, an abbreviated version of the COPE Inventory [64]. We used the Polish version adapted by Juczyński and Bulik [65]. The questionnaire includes 28 items on 14 subscales, which might be clustered into adaptive or maladaptive strategies. The adaptive strategies include *Active coping, Emotional support, Use of informational support, Positive reframing, Planning, Humor, Acceptance, Religion*. In turn, *Self-distraction, Denial, Substance use, Behavioral disengagement, Self-blame*, and *Venting* are considered maladaptive strategies. Cronbach’s alpha were as follows: *Active coping* α = 0.65; *Planning* α = 0.88; *Positive reframing* α = 0.68; *Acceptance* α = 0.67; *Humor* α = 0.83; *Religion* α = 0.94; *Use of emotional support* α = 0.92; *Use of instrumental support* α = 0.87; *Self-distraction* α = 0.85; *Denial* α = 0.76; *Venting* α = 0.70; *Substance use* α = 0.59; *Behavioral disengagement* α = 0.70; *Self-blame* α = 0.91.

#### 2.2.4. Body Composition Analyzer

In order to gather the participants’ anthropometric data, a BC-601 Tanita Segmental Body Composition Monitor produced by Tanita Corporation Japan was used. The importance of taking visceral fat into account, and not just body mass index (BMI), is increasingly emphasized [66,67]; therefore, we also considered visceral fat, despite basing the initial inclusion criteria on BMI. For adults, the analyzer allows the measurement of indices of obesity adjusted for muscle mass content, fat percentage (%BF), recommended daily energetic intake, basal metabolic rate, metabolic age, bone mass, and visceral fat content [68]. The age of participants was taken into account when determining body-fat status (i.e., overfat, healthy, or underfat) [69].

### 2.3. Statistical Analysis

The analyses were performed in Python 3.8.5 programming language (Python Software Foundation, Fredericksburg, VA, USA), using JupiterLab 2.2.6 as the computation environment. The following Python libraries were used:

- pandas 1.1.3
- scipy 1.5.2
- numpy 1.19.2
- pingouin 0.3.9

Test data were contained in pandas.DataFrame. DataFrame is a two-dimensional, size-mutable, potentially heterogeneous tabular data type. Its structure contains labeled axes (rows and columns). Arithmetic operations align on both row and column labels [70]. It can be considered a mathematical database. This study used DataFrame to index and align data. Each row contained all data collected from a single participant.

Student’s t-test was performed using scipy.stats.ttest_ind function. Mann–Whitney U test was performed using scipy.stats.mannwhitneyu function. Spearman’s Rho correlations were calculated using scipy.stats.spearmanr function. Mediation analysis was performed using pingouin.mediation_analysis function. Z-Score was calculated by executing pandas.DataFrame.apply(numpy.mstats.zscore).

Validity of group selection was determined by using mean analysis and Student’s t-tests on weight, visceral fat, fat percentage, BMI, age, height, and desired weight reduction. Moreover, Mann–Whitney U was used to determine differences between groups for PSQ, HSQ, and Brief COPE Scale. Spearman’s Rho correlations were calculated between the fat index and PSQ, visceral fat and PSQ, desired weight reduction in percent, and PSQ. It was also used to determine the correlation between fat percentage and Brief COPE Scale, visceral fat and Brief COPE Scale, desired weight reduction in percent, and Brief COPE Scale. Bias-correct, nonparametric, bootstrap mediation was used to determine the relationship between humor styles and coping strategies with stigma as a mediator.

## 3. Results

### 3.1. Anthropometric Differences

First, we wanted to verify if the selection of respondents was adequate; thus, we examined the significance of differences between groups distinguished on the basis of whether the women were overfat or at healthy fat levels in terms of all variables included in the research (Table 1).

The two groups differed in terms of fat percentage (further described in the tables as %BF), visceral fat, weight, and BMI. The percent of reduction of body weight in order to attain one’s dream weight differed between groups. The study groups did not differ in terms of height and age.

#### 3.1.1. Perceived Stigmatization

The groups differed significantly in terms of perceived stigmatization (*t*(125) *=* 2.69, *p =* 0.009), which derives from the differences on the confused/staring behavior (*t*(125) *=* 2.93, *p =* 0.004) and hostile behavior (*t*(125) *=* 3.76, *p* < 0.001) subscales. A correlation was observed between the percentage and overall score for perceived stigmatization in both groups (Table 2).

Women with overfat perceive stigmatization, including staring and aggressive behaviors more often than women whose weight is within norms. It can also be observed that women whose weight is average do not experience a lack of positive behavior even if their levels of fat are high.

#### 3.1.2. Coping with Stress Situation and Sense of Humor

The results indicate that the groups did not differ in terms of strategies of coping with stressful situations, except in terms of using humor to cope with stress (*t*(125) *=* 2.8, *p =* 0.007). At the same time, due to the correlation of %BF and visceral fat with coping strategies, women in the overfat group were more likely to use Planning, denial, and use of informational support as ways to cope with stressful situations (Table 3). 

Moreover, the bigger the difference between the current weight and the goal (ideal) weight, the more frequently the women with overfat used self-blame and behavioral disengagement, while women with healthy fat leaned towards denial and away from religion.

The groups did not differ significantly in terms of the style of humor used. However, there was a correlation between fat percentage and aggressive humor (*r* = 0.32, *p =* 0.020) and self-defeating humor (*r* = 0.31, *p =* 0.023) in the obese group. 

#### 3.1.3. Perceived Stigmatization as a Mediator between Humor Styles and Coping with the Stress 

**Figure 1:**
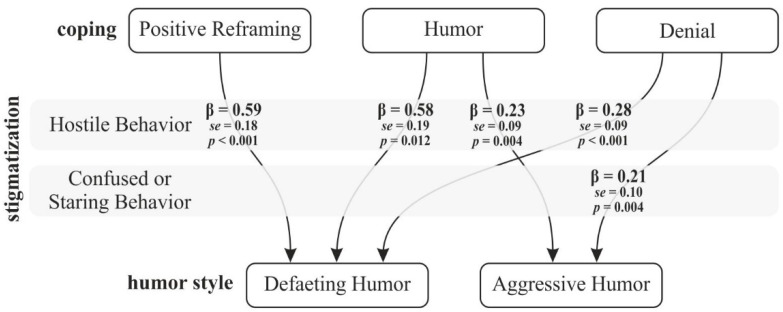
Perceived stigma as a mediator between humor style and ways of coping with stress in group women with overfat.

In investigating the hypothesis that perceived stigmatization mediates the relationship between sense of humor and strategies of coping with stress, a mediating effect of hostile behavior was observed in group women with overfat, only for the relationship between self-defeating humor and humor as a way to cope with stress (Figure 1).

At the same time, we observed the influence of stigmatization on the relationship between styles of humor and ways of coping with difficult situations, only in the groups of women with overfat. The strongest effect was observed in the relationships where hostile behavior was the mediator—between positive reappraisal and self-defeating humor as well as between humor and self-defeating humor in women with overfat. Moreover, perceived stigmatization influenced the relationship between humor as a way to cope with stress and its maladaptive styles. No statistically significant effects were observed in the group of women with healthy fat. This relationship did not occur in the group of women with healthy fat.

## 4. Discussion

Our results partially confirmed hypothesis 2. Perceived stigmatization mediated the relationship between the style of humor and ways of coping with stressful situations only in the group of women with overfat. Hypothesis 1 was not confirmed in our study.

### 4.1. Anthropometric Variables

All the participants in this study desired to reduce their body mass. This suggests that none of the participating women were satisfied with their bodies. This is in contrast to the results of Annis et al. [29], who found that, in a sample of 165 women, women with overweight reported greater dissatisfaction with their appearance. Similarly, a study by Siervo et al. [71] found that dissatisfaction with one’s body and the desire to reduce one’s body mass were greater among women with obesity than those without. Dissatisfaction with one’s own body may accompany women throughout their lives, starting in the earliest years. However, this can depend on culture, as Asian American respondents have been found to not fit this pattern—most commonly reported problems in this group are weight gain, desire to lose weight, and mild dissatisfaction with one’s body [72]. 

Interestingly, for men, body fat is not necessarily associated with aspirations to change one’s weight (either increasing or decreasing one’s body mass) [73]. In one sample of 996 male participants aged 19–26, 29.72% wanted to decrease their body mass and 53.21% wanted to increase it, by 8.57 kg on average (*SD =* 5.85). On the other hand, the better their assessment of their physical fitness is, the lower their declared desire is to reduce weight. This is in line with research by Franzoi [74], who emphasized that understanding one’s body as an object is more characteristic of women, who tend to assess their body parts separately, while men tend to understand their bodies as a process, where the body’s functionality is what is assessed. Franzoi et al. [15] also observed that men are more self-hopeful, and women are more self-critical with regard to their bodies. Particularly when comparing themselves to others, women are more likely to compare themselves to other women who they perceive as more attractive.

### 4.2. Obesity and the Subjective Perception of Stigmatization 

A person with obesity is unable to hide their condition, which puts them at risk of being judged by people around them, even if the sense of being judged is just a subjective perception. In our study, women with overfat considered themselves more stigmatized than women whose weight was average, and the associated discomfort was significantly related to the sense of “being fat,” because the larger the difference between current weight and the desired weight was, the more stigmatization the women perceived. The role of body mass for perceived stigmatization, as we can see in our results, is also visible in cross-cultural studies: Polish women felt less stigmatized than Vietnamese women if their body mass was not taken into account [32]. Interestingly, lack of friendly behaviors from the environment, that is to say, a lack of compliments, as a component of stigmatization, influences perceptions of one’s body in both cultures. In our research, women with healthy fat do not focus on compliments understood as friendly behaviors. An experiment by Blodorn et al. [75] had participants make a speech describing why they would make a good date and were told that a potential partner would either see or hear a recording of them making the speech. It turned out that women with higher body mass had higher expectations of social rejection when they would be seen (versus unseen). Obesity is stigmatized from the earliest years of life [76,77].

Our research showed that there is a relationship between the objective dimensions of a person’s body and perceived stigmatization. In our previous research, obese women perceived greater stigmatization through hostile behaviors than women of normal weight or women with skin diseases; they also assessed their quality of life as lower [11]. It is in accordance with a study by Annis et al. [29], which showed that body image dissatisfaction/distress, dysfunctional appearance investment, and overweight preoccupation are more associated with stigmatizing experiences at various ages in currently overweight women than in formerly overweight women. The authors also noted the phenomenon of “phantom fat”, i.e., an effect where an individual still perceives themself as obese even after losing weight, which is also associated with stigmatization. This can indicate that the environment plays an important role in perceived stigmatization. Participants in a two-week-long study by Vartanian et al. [30] described their experiences with weight stigma. Over this period, 91% of participants experienced stigmatization at least once, and the overall frequency of stigmatizing experiences was not associated with BMI. It seems interesting that the most frequent sources of stigmatization, just after strangers, were those closest to the participants—spouses, partners, friends, and parents. Interestingly, media and advertisements were causes of perceived stigmatization in 54% of respondents. 

Despite the fact that all participating women wanted to lose weight, it seems that in the group of women who were not overfat, this was not related to the experience of stigmatization. Lack of compliments and positive behaviors toward women who are not obese were not perceived as problems, even if they had a high body fat percentage. This is in contrast to the data gathered by Lipowska et al. [32], who found that independently of body mass, lack of perception of friendly behaviors is considered an important element of stigmatization by young women, and compliments play an important role in building self-esteem.

### 4.3. The Role of Humor in Dealing with Obesity Stigma 

There exists a social belief that “fat” individuals are funnier, more entertaining, and merrier [78]. As shown by our study, women with overfat more often use humor to reduce stress, especially in situations where they feel stigmatized. Interestingly, this humor is often aimed at themselves, as self-depreciation. At the same time, obese individuals are often the objects of jokes and are used as a source of humor in entertainment media; they are thus an object of ridicule, and references to one’s own weight are typically met with laughter [42].

Obesity, as a stigmatizing condition, to a large extent exposes an individual to psychological stress, and it is a difficult situation one needs to cope with. As a chronic disease, it is long-term and generates the need to cope with it and its consequences.

Research by Rahnavard et al. [39] on 24 obese women waiting for Slevee surgery who experienced weight-related stigmatization indicate that in order to deal with this stigma, respondents used social resistance, passivity, psychological problems and hysteria, extreme denial of self-body image, social isolation, and ignorance of what others say. Research by Himmelstein [79] suggests that in people experiencing stigma related to weight, stigma-specific coping responses mediated the relationship between experienced weight stigma and all investigated health indicators, although the indirect effects of weighting on health differed with coping strategies.

Assuming that obesity, as a source of anxiety, leads to increased tension and that women with overfat experience stress associated with attractiveness and appearance more often than those whose weight is within norms [35], we hypothesized that strategies for coping with stress would be different for the two groups of participants. However, the results did not support this hypothesis. 

Our research shows that obese women, especially when they want to lose a significant amount of weight, use maladaptive coping strategies. This is in line with the studies by Varela et al. [80], in which people with excess weight more often used passive strategies of coping with stress, such as self-criticism, wishful thinking, social withdrawal, and emotional eating, and restrained eating. These lead to unhealthy eating behavior and are more likely to contribute to the emergence and maintenance of a high BMI. Brytek-Matera [23] showed that women with obesity, focusing on their own emotional states and thus not taking action to solve the existing problem, avoid situations that have led to emotional tension.

We expected that sense of humor as a way to cope with difficulties would be characteristic of women with overfat, and the results supported this. The originators of the concept of humor styles [56] indicate that humor may have an adaptive character, but that it can also be harmful and maladaptive. Thus, it is not the sense of humor in itself but rather the ways one uses it and the goals one has when using it that are important for understanding its role in everyday functioning. Maladaptive styles of humor are associated with aggression and being snide. Aggressive humor is directed outwardly; it is associated with raising one’s status and mood by demeaning and ridiculing other people, making fun of them [56,62]. Self-defeating humor is based on the need for approval through paying the price of ridiculing oneself. It is expressed through attempts to make people laugh by telling self-ridiculing stories [81]. Oftentimes, an individual who uses this kind of humor can be perceived as funny or witty (e.g., “the class clown”). At the same time, they often hide their emotional needs and have low self-esteem, and the humor is used as a form of defensive denial or for ridiculing one’s shortcomings [52,56,81,82]. According to this view, as also shown by our study, when women with overfat face threatening situations where they perceive behaviors that are hostile toward them, using humor to cope with stress, they select maladaptive styles of humor.

It can be thus supposed that it is easier for a woman with overfat to laugh at herself and her situation than to be left with a sense of stigmatization and rejection. Interestingly, a cross-cultural study [83] found that Polish men are characterized by higher levels of the self-defeating humor style than Polish women. In this same study—on 8,361 participants from 28 countries—men were characterized by higher levels of self-enhancing humor, self-defeating humor, and aggressive humor. Affiliative humor did not differ between the genders in any of the 28 countries. Affiliative humor and self-enhancing (i.e., adaptive) styles of humor in stigmatizing situations did not affect the styles of coping with stress among the participants of our study. 

Women who desire to lose weight often blame themselves or give up when facing difficulties [26]. This is often associated with blaming oneself for the lack of results of attempts to lose weight; they feel guilty because their efforts did not effectively change their situation. They need support from loved ones and institutions. At the same time, research shows that even healthcare providers, including medical students, can stigmatize obesity. In an interventional study by Kushner et al. [84] medical students initially revealed stereotypical thinking regarding obesity and their lack of empathy toward patients with this condition. Only after a session in which they entered the role of an obese patient did their assessment of obese patients significantly change. In a follow-up a year later, and it turned out that the students still showed empathy toward obese patients; however, stereotypes returned to the levels from the first measurement. Phelan et al. [85] conducted a narrative review of the literature regarding research on the effects of stigma on the healthcare received by people who are obese. They observed that attitudes toward obesity as a risk factor for health exacerbated and masked negative attitudes toward obese individuals, while health sector workers often perceive obesity as an avoidable risk factor. This shows how many stigmatizing situations are experienced by people with overfat on a daily basis, which requires them to launch coping strategies to function in society. Such behavior does not support the prevention or therapy of obesity. This is because treating obesity does not depend solely on body mass reduction. Support by close ones may expedite, strengthen, and make the effects of weight loss therapy more lasting. Similar conclusions were drawn from a study by Powers et al. [86], in which college women who had themselves chosen to lose weight reported greater weight loss if they had the support of family and friends. The positive effects of support persisted even if the support itself decreased with time.

### 4.4. Limitation and Future Perspectives

The limitation of this study is undoubtedly the number of women with overfat. Despite the fact that the number of people with overfat is constantly increasing, not all women invited to the study agreed to anthropometric tests and measurements. The sample presented in this research limits the number and depth of analysis that can be performed. While we can correlate anthropometric variables to humor and coping strategies, only experimental and longitudinal studies may establish clear causation. It is worth continuing the study to assess whether similar results will be obtained in a larger group. This research focuses only on a group of young women due to the social disapproval of excess body fat in this group, but it is worthwhile to include also young men with overfat in the research in order to verify the role of gender differences. Moreover, the method of participants recruitment—a purposive sampling—might be a limitation of the study. Moreover, the results of our study cannot be generalized to all overfat women because of the sample size.

It is also worth assessing whether the role of a sense of humor or the specificity of coping with the stigma of obesity is related to gender. We also find it worthwhile to expand the study with questionnaires focusing on coping with weight-related stigma to examine the above problems from a more holistic perspective. 

## 5. Conclusions

Our research confirmed that the sense of stigma affects the relationship between the styles of sense of humor and the ways of coping with difficult situations in women with overfat. Results indicate that, when perceiving hostile behaviors toward themselves and using humor as a coping strategy, women with overfat select maladaptive styles of humor (i.e., self-defeating and aggressive styles). Moreover, a sense of humor as a strategy of coping with a stressful situation was more often used by women with overfat. 

Despite the fact that women with overfat felt stigmatized much more often than women with healthy fat, it was surprising that none of the participants were satisfied with their body mass. At the same time, among women without obesity, a lack of compliments was not treated as a problem, even if they had high body fat.

This study highlights how perceived stigmatization affects women with overfat and also how it is implicated in their behaviors toward themselves and others. Using strategies for coping with stress and using humor in a destructive manner affect the stigmatized woman herself and her environment and relationships with those who are close to her. 

The data about weight-related stigmatization and its consequences for women with obesity found in this study may have great importance for the practice of general practitioners, psychiatrists, pedagogues, and other specialists who support the health and development of women (from the earliest years). It is important to have conversations about the consequences of discrimination to support people who are obese both in the process of weight loss and in accepting their circumstances. The social acceptance of obesity stigma does not support the processes of weight loss and recovery in individuals affected by this condition. 

It is important to lower the levels of stigmatization through the following strategies:

(a) Prevention, starting at the earliest years, at school and at home, in terms of education about health, physical activity, and sugar and fat metabolism;
(b) Providing support and comprehensive help for individuals who have excess weight, including psychological and medical help;
(c) Providing support for families of obese individuals and teaching appropriate behaviors with regard to improving the health and quality of life of the affected individuals.

Stigmatization does not help people “to not be fat”; instead, it fosters a sense of blame and lack of agency about one’s appearance, potentially leading to increased unhealthy eating behaviors and thus to weight gain in some individuals. One’s way of thinking has a significant influence on the course of obesity and the effects of the prevention and therapy thereof.

## Figures and Tables

**Table 1:** Descriptive statistics and differences between groups.

Feature/Index	Healthy Fat (*n* = 73)				Overfat (*n* = 54)				Differences (*df* = 125)	
	*M*	min	max	*SD*	*M*	min	max	*SD*	*t*	*p*
%BF	28.82	19.60	37.40	3.60	43.44	29.80	52.80	4.91	18.52	<0.001
Visceral Fat	1.89	1.00	4.00	0.79	8.63	5.00	15.00	2.47	19.29	<0.001
Weight	61.36	52.20	71.00	5.93	98.17	64.50	143.00	15.14	16.93	<0.001
Heigh	167.55	148.00	182.00	6.14	167.37	153.00	180.00	5.36	0.17	0.863
Age	25.41	19.00	30.00	2.79	26.19	21.00	30.00	2.62	1.60	0.110
BMI	21.84	18.70	24.50	1.63	35.01	30.10	45.70	4.15	22.11	<0.001
Desired weight reduction in %	8.82	0.55	17.46	4.37	27.41	10.21	43.96	8.97	14.04	<0.001

**Table 2:** Correlations of PSQ scales with %BF, visceral fat, and desired weight reduction (in %).

PSQ Scales	%BF		Visceral Fat		Desired Weight Reduction in %	
	Overfat	Healthy Fat	Overfat	Healthy Fat	Overfat	Healthy Fat
Absence of Friendly Behavior	0.05	−0.34 **	−0.01	−0.18	0.04	−0.17
Confused/Staring Behavior	0.38 **	−0.14	0.34 *	−0.20	0.42 **	0.08
Hostile Behavior	0.32 *	−0.11	0.25	−0.06	0.17	0.00
Total	0.38 **	−0.28 *	0.31 *	−0.21	0.31 *	−0.06

Note: * *p* < 0.05, ** *p* < 0.01.

**Table 3:** Table of correlation between Brief COPE Scale and %BF, visceral fat, and desired weight reduction in%.

Brief Cope Scales	%BF		Visceral Fat		Desired Weight Reduction in%	
	Overfat	Healthy Fat	Overfat	Healthy Fat	Overfat	Healthy Fat
Active Coping	0.22	−0.04	0.23	−0.02	0.12	−0.21
Planning	0.30 *	−0.06	0.32 *	0.03	0.20	−0.01
Positive reframing	0.18	−0.02	0.20	−0.04	0.24	−0.25 *
Acceptance	−0.03	−0.16	−0.01	−0.15	−0.01	−0.05
Humor	0.02	−0.04	0.07	−0.20	0.06	−0.25 *
Religion	0.07	−0.01	−0.07	−0.06	0.00	−0.28 *
Use of emotional support	0.22	−0.05	0.14	−0.01	0.04	0.03
Use of informational support	0.29 *	−0.15	0.29 *	−0.15	0.13	0.04
Self-distraction	−0.03	0.21	−0.06	0.09	0.27 *	0.11
Denial	0.32 *	0.06	0.37 **	0.07	0.26	0.28 *
Venting	−0.04	0.02	0.05	0.02	0.17	0.13
Substance Use	−0.06	0.05	−0.18	−0.03	0.00	0.10
Behavioral disengagement	0.00	−0.23 *	0.06	−0.23	0.35 **	0.19
Self-blame	0.16	0.02	0.15	0.09	0.40 **	0.13

Note: * *p* < 0.05, ** *p* < 0.01.

## Data Availability

The data from this study are available from the corresponding author upon request.
